# Hepatitis E in Long-Term Travelers from the Netherlands to Subtropical and Tropical Countries, 2008–2011

**DOI:** 10.3201/eid2406.171513

**Published:** 2018-06

**Authors:** Floortje Elfrink, Femke W. Overbosch, Janke Schinkel, Gerrit Koen, Gerard J.B. Sonder

**Affiliations:** Public Health Service, Amsterdam, the Netherlands (F. Elfrink, F.W. Overbosch, G.J.B. Sonder);; National Coordination Centre for Traveller’s Health Advice, Amsterdam (F. Elfrink, G.J.B. Sonder);; Academic Medical Center, Amsterdam (J. Schinkel, G. Koen, G.J.B. Sonder)

**Keywords:** hepatitis E, hepatitis E virus, HEV, viruses, travel, long-term travelers, prospective study, attack rate, incidence, the Netherlands, subtropical countries, tropical countries

## Abstract

Hepatitis E virus (HEV) is a common cause of acute viral hepatitis. Virus genotypes 1 and 2 infect humans in developing countries by the fecal–oral route. To assess attack rates and disease incidence for travelers, we prospectively studied 604 long-term travelers to subtropical and tropical countries. Participants donated blood samples pretravel and posttravel and kept a diary. A total of 89/604 (15%) pretravel samples were positive for HEV IgG by ELISA, suggesting previous HEV infection. Seroconversion for HEV was found for 19/515 travelers (attack rate 3.7%, incidence 1.8 cases/1,000 person-weeks). We believe there is a substantial risk for acquiring HEV infection among long-term travelers. Although HEV infection does not seem to be a major problem in this healthy cohort, hygienic measures should be stressed in all pretravel health advice, particularly for pregnant women and immunocompromised travelers who are at risk for severe disease.

Hepatitis E virus (HEV) is a common cause of acute viral hepatitis worldwide (*1*). There are 4 genotypes of HEV. Genotypes 1 and 2 infect humans in developing countries in areas with poor sanitation; transmission occurs through the fecal–oral route, causing occasional large outbreaks or frequent sporadic cases. Genotype 1 is found in Asia and Africa, and genotype 2 is found in Mexico and Africa. Genotypes 3 and 4 are transmitted zoonotically from animal reservoirs in industrialized and developing countries, mainly through consumption of uncooked or undercooked meat, and are responsible for sporadic cases of disease (*1*). In the Netherlands, genotype 3 is endemic in pigs and responsible for cases in humans.

The mean incubation period for hepatitis E is 40 days (range 15–60 days). Symptoms range from subclinical to fulminant and include fever, fatigue, loss of appetite, nausea, vomiting, abdominal pain, jaundice, joint pain, and hepatomegaly (*1*).

Hepatitis E is usually a self-limiting disease. The mortality rate for fulminant hepatitis is 0.5%–4%. Pregnant women, immunosuppressed persons, and persons with preexisting liver disease are at risk for severe hepatitis E. However, fulminant liver disease in immunocompetent persons has also been reported (*2*). Mortality rates for hepatitis E caused by genotype 1 for pregnant women are 20%–25% (*3**,**4*).

A previous HEV infection is characterized by the presence of specific IgG and is assumed to protect against reinfection. There is cross-neutralization among all genotypes (*5*).

Travelers from industrialized countries to developing countries are assumed to be at risk for acquiring an HEV infection through the fecal–oral route (genotypes 1 and 2). A vaccine against HEV is available only in China (*4*).

A study that included data in the GeoSentinel surveillance network for returned travelers with infectious gastrointestinal diseases during 1996–2005 reported a proportionate HEV illness rate of 1.2 cases/1,000 ill returned travelers (*6*). A recent case report identified a nonpregnant immunocompetent traveler who returned to Canada from India and was given a diagnosis of HEV infection, in whom fulminant liver failure developed (*2*). A study in Israel of 4,970 ill returning travelers during 1997–2012 reported 49 (1%) with acute hepatitis (32 cases were enterically transmitted): 19 travelers were given a diagnosis of hepatitis E, of whom 16 contracted their cases on the Indian subcontinent (*7*). The estimated risk for acquiring HEV for this study was 3.2 cases/100,000 travelers.

A prospective study of 1,206 short-term travelers from the Netherlands to subtropical and tropical countries (*8*), a prospective study of 105 long-term backpackers in Israel (*9*), and a prospective study of American missionaries (*10*) showed no seroconversions for HEV. Another prospective study of 356 short-term travelers from the United States reported 4 (1.7%) seroconversions (*11*). However, because seroconversions were found only for samples obtained 6 months after return of travelers and not in samples obtained 6 weeks posttravel, HEV might have been contracted after their return.

To our knowledge, no recent prospective studies of long-term travelers have been conducted. Because the risk for hepatitis E in subtropical and tropical countries might have increased, and the sensitivity of ELISAs for diagnosing HEV infection has improved over the past decade (*3*), we determined the incidence and risk factors of acquiring hepatitis E among long-term travelers (12–52 weeks) from the Netherlands to subtropical and tropical countries.

## Methods

### Study Population and Design

This study was conducted as part of a larger, prospective, monocenter study of immunocompetent travelers >18 years of age who visited the Public Health Service travel clinic in Amsterdam, the Netherlands, during December 2008–September 2011. All clients planning to travel to subtropical or tropical countries for 12–52 weeks were invited to participate. Subtropical and tropical countries were defined as those with moderate-to-high risk for hepatitis A according to the World Health Organization (*12*). All participants consulted a nurse or physician specialized in travel medicine, and oral and written information was provided about how to avoid travel-related diseases. The study was approved by the Medical Ethics Committee of the Academic Medical Center in Amsterdam.

At their return, travelers were asked additional questions regarding behavior during travel, including drinking unboiled tap water or water from natural sources. Pretravel written informed consent was obtained, and travelers were interviewed by a nurse or physician about travel purpose, travel duration, planned destination(s), and demographic details.

Participants were given a digital thermometer (Huikeshoven Medical, Tiel, the Netherlands) and asked to record their temperatures if they felt feverish while traveling. Travelers kept a structured, weekly travel diary until 2 weeks after return and recorded their itinerary, symptoms, and physician visits while ill. Predefined symptoms that could be related to hepatitis E were fever and vomiting. It was also possible to report other complaints. Diaries were completed on paper or digitally. Travelers received a weekly email reminder and were seen 2–6 weeks after return. Blood samples were obtained before and after travel. The pretravel sample was tested only if the posttravel sample tested was positive for antibodies against HEV.

Primary regions visited were grouped into regions according to the classification of the United Nations Country Classification with some modifications. For our study, Oceania included only Melanesia, Micronesia, and Polynesia and was merged with Southeast Asia because New Zealand and Australia matched exclusion criteria for participating. Latin America was divided into South America and the Caribbean/Central America.

### Laboratory Methods

Blood samples were immediately stored at 6°C, centrifuged, and frozen at −80°C. We tested serum samples for HEV IgG by using an ELISA (Wantai Biologic Pharmacy Enterprise, Beijing, China), according to the manufacturer’s instructions. This assay had a reported sensitivity of 98% but does not discriminate between different virus genotypes. If HEV IgG was detected in a posttravel sample, pretreatment samples were also tested. Presence of HEV IgG in a pretravel sample was regarded as evidence of previous HEV infection. A recent infection was defined as a positive posttravel sample and a negative pretravel sample.

### Data Analysis

We calculated risk factors for previous HEV infection by using SPSS version 19.0 (IBM, Armonk, NY, USA) to obtain prevalence, univariable and multivariable prevalence ratios (PRs), and 95% CIs by means of logistic regression modeling. A p value <0.05 was considered statistically significant. All variables with p values <0.10 by univariable analysis were included in a multivariable model.

We calculated the attack rate of a recent HEV infection by dividing the number of seroconversions by the total number of participants still at risk for infection (i.e., all travelers who did not have HEV antibodies pretravel). We also calculated incidence rates by dividing the number of seroconversions by the total number of travel weeks of travelers still at risk. Person-time denominators for seroconversion were divided in half, assuming that the infection occurred halfway through travel.

We used univariable Poisson regression models to examine the effect of covariates (sex, age, travel purpose, primary destination, hospital admission) on seroconversion. Variables with p values <0.10 in univariable analysis were included in multivariable analysis. Outcomes were expressed as incidence rate ratios with 95% CIs. A p value <0.05 was considered statistically significant.

## Results

### Study Population

During December 2008–September 2011, a total of 685 persons who intended to travel to subtropical and tropical countries for 12–52 weeks provided informed consent. Of these persons, 81 (12%) were excluded after completion of the study: 42 had their travel arrangements changed and no longer met the study criteria, 38 were lost to follow up, and 1 did not provide a posttravel blood sample. The remaining 604 persons formed the study population.

Median age of the study population was 25 years (interquartile range [IQR] 23–29 years), ≈66.6% were female, and 20% had never been to subtropical or tropical regions. Tourism was the main purpose for traveling (62.9%). Median interval between the first sample and departure was 38 days (IQR 20–55 days). Median interval between return and the second blood sample was 25 days (IQR 21–33 days).

### Previous HEV Infection

A total of 89 of 604 persons were positive for HEV pretravel and posttravel, which indicated previous HEV infection, for a pretravel seroprevalence rate of 14.7% (Table 1). Univariate analysis indicated that previous HEV infection showed a positive correlation with older age, a nonwestern origin, and a history of travel to subtropical or tropical regions. Multivariate analysis showed that age, travel history, and nonwestern origin remained major predictors for previous HEV infection.

**Table 1:** Characteristics of 604 travelers who visited a travel clinic for pretravel advice and prevalence of previous HEV infection, the Netherlands, December 2008–September 2011*

Characteristic	Travelers	HEV IgG positive pretravel	Univariable analysis			Multivariable analysis	
			PR (95% CI)	p value		PR (95% CI)	p value
Total	604	89 (14.7)					
Median age, y (IQR)	25 (23–29)	26 (23–30)	1.0 (1.01–1.07)	**0.002**		1.0 (1.01–1.06)	**0.01**
Sex							
F	389 (64.4)	54 (13.9)	1.0	0.43			
M	215 (35.6)	35 (16.3)	1.2 (0.76–1.92)				
Region of birth							
Western (the Netherlands), n = 563	590 (97.7)	84 (14.2)	1.0	**0.034**		1.0	**0.03**
Nonwestern	14 (2.3)	5 (35.7)	3.4 (1.10–10.23)			3.6 (1.5–11.28)	
Previous travel to subtropical region							
No	122 (20.2)	7 (5.7)	1.0	**0.003**		1.0	**0.01**
Yes	482 (79.8)	82 (17.0)	3.4 (1.5–7.5)			2.9 (1.27–6.45)	

*Values are no. (%) except as indicated. Bold indicates statistical significance. HEV, hepatitis E virus; IQR, interquartile range; PR, prevalence ratio.

### HEV Infection Acquired during Current Travel

IgG seroconversion was found for 19/515 travelers, resulting in an attack rate of 3.7% and an incidence of 1.8 (95% CI 1.1–2.8) per 1,000 person-weeks. We obtained characteristics, attack rates, and incidence for recent HEV infections (seroconversions) (Table 2). At return, 32% (163/510) of participants reported they had used unboiled or untreated tap water for consumption, 19 did not remember, and 5 had missing results. The remaining 328 travelers did not consume unboiled or untreated water. Six persons who showed seroconversion reported having drunk unboiled or untreated tap water; 1 person had a missing result. Logistic regression of characteristics tested did not identify any major risk factors for acquiring HEV infection during travel.

**Table 2:** Attack rates and incidence of seroconversions in HEV antibody levels for 515 long-term travelers to subtropical and tropical countries, the Netherlands, December 2008–September 2011*

Characteristic	Travelers at risk	HEV seroconversions	Attack rate, % (95% CI)	Person-weeks of travel	Incidence/1,000 person-weeks (95% CI)		
						Univariable analysis	
						IRR (95% CI)	p value
Total	515	19	3.7 (2.4–5.7)	10,715	1.8 (1.1–2.8)		
Median age, y (IQR)	25 (23–29)	26 (22–31)					
Sex							
F	335 (65)	9	2.7 (1.4–5.0)	6,795	1.3 (0.7–2.5)	1.0	0.16
M	180 (35)	10	5.6 (3.0–9.9)	3,920	2.6 (1.4-4.7)	1.9 (0.8–4.7)	
Region of birth							
Western	506 (98.3)	19	3.8 (2.4–5.8)				
Nonwestern	9 (1.7)	0	NA				
Purpose of travel							
Holiday	324 (62.9)	10	3.1 (1.7–5.6)	6,556	1.5 (0.8–2.8)	1.0	0.54
Work or study	184 (35.7)	8	4.3 (2.2–8.3)	3,970	2.0 (1.0–4.0)	1.3 (0.5–3.3)	
VFR	7 (1.4)	1	14.3 (2.6–51.3)	1,885	5.3 (0.9–29.4)	3.5 (0.4–27.0)	
Primary region of travel							
Southeast Asia and Oceania	172 (33.4)	5	2.9 (1.2–6.6)	3,543	1.4 (0.6–3.3)	1.0	0.57
South America	148 (28.7)	5	3.4 (1.5–7.7)	3,211	1.6 (0.7–3.6)	1.1 (0.3–3.8)	
Sub-Saharan Africa	94 (18.3)	2	2.1 (0.6–7.4)	1,972	1.0 (0.3-3.7)	0.7 (0.1–3.7)	
Southern Asia	47 (9.1)	3	6.4 (2.2–17.2)	898	3.3 (1.1–9.8)	2.4 (0.6–9.9)	
Central America and Caribbean	37 (7.2)	3	8.1 (2.8–21.3)	754	4.0 (1.4–11.6)	2.8 (0.7–11.8)	
Asia, other	17 (3.3)	1	5.9 (1.0–27.0)	336	3.0 (0.5–16.7)	2.1 (0.2–18.1)	
Travel duration, wk							
12–16	189 (36.7)	6	3.2 (1.5–6.8)	NA			
17–24	190 (36.9)	7	3.7 (1.8–7.4)	NA			
25–52	136 (26.4)	6	4.4 (2.0–9.3)	NA			
Hospital admission							
No	484 (94)	18	3.7 (2.4–5.8)	10,032.5	1.8 (1.1–2.8)	1.0	0.84
Yes	31 (6)	1	3.2 (0.6–16.2)	682.5	1.5 (0.3–8.2)	0.8 (0.11–6.11)	

*Values are no. (%) except as indicated. HEV, hepatitis E virus; IQR, interquartile range; IRR, incidence rate ratio; NA, not applicable; VFR, visiting friends and relatives.

### Signs and Symptoms in Travelers Showing Seroconversion

A total of 215 (42%) of 515 travelers reported vomiting during their trip, and 35% (180/515) reported fever at least once. Nine of 19 travelers showing seroconversion reported >1 nonspecific symptoms possibly associated with HEV infection: 2 participants reported fever, 3 reported vomiting, 1 reported vomiting and fatigue, 2 reported vomiting and fever, and 1 reported abdominal pain and nausea.

A total of 31 (6%) of 515 participants were admitted to a hospital while abroad, of whom 1 person who showed seroconversion, was admitted because of symptoms of fever and dehydration caused by diarrhea. Jaundice, dark-colored urine, and light-colored stool were not reported as other complaints in the diary.

## Discussion

In this prospective study of long-term travelers from the Netherlands to subtropical and tropical countries, we found a substantial hepatitis E attack rate of 3.7% and an incidence of 1.8 cases/1,000 person-weeks. Results were obtained by using an HEV IgG ELISA and were higher than those in the 4 previous prospective studies of travelers (*8**–**11*).

The relatively high HEV seroconversion rate we found compared with those for previous prospective studies could be explained by an increase of hepatitis E incidence in developing countries over time. However, our results probably reflect improved sensitivity of currently available tests compared with those used in these previous studies. A combination of these factors is possible. Therefore, comparison of results of previous prospective and seroprevalence studies with those of our study should be interpreted with caution. We also found no major risk factors for acquiring HEV infection during travel.

Two nonprospective studies reported that hepatitis E is associated with travel to southern Asia (*7**,**13*). We found higher attack rates and incidences for southern Asia, other regions of Asia, and Central America than for Africa, Southeast Asia, and South America, but this finding did show a major difference.

In 2 other studies, travelers visiting friends and relatives were found to be at greater risk than tourist travelers for infectious diseases such as typhoid fever (*6*) and hepatitis E (*14*). Our study showed a higher attack rate (14%) and incidence (5.3 cases/1,000 person-weeks) for travelers visiting friends and relatives than for persons traveling for tourism or work/study, but this association was not strong. This finding could be caused by the small numbers of travelers visiting friends and relatives.

The pretravel seroprevalence of 15% we found was higher than the 2% found in the study of travelers from the Netherlands conducted during 2006–2007 (*8*) and the 6% found in the study of Boston, Massachusetts, USA, area travelers conducted during 2009–2010 (*15*). However, seroprevalence in this study was lower than the 27% found in the study of blood donors from the Netherlands conducted during 2011 (*16*) and in the population of Amsterdam during 2004 (*17*). Although these differences should also be interpreted with caution, there are several possible explanations for the differences in prevalence between studies.

The major difference in sensitivity between different assays could be an explanation for the higher prevalence we found than the prevalence of 2% found in the previous prospective study among travelers from the Netherlands. The test we used in our study was the same test used in the study of blood donors from the Netherlands (*16*) and for the population of Amsterdam (*17*). However, because HEV immunity increases with age and depends on ethnicity, the difference in characteristics between the different study groups could explain why we found a prevalence of 15% rather than 27%. Our study population was composed of mostly young persons of western origin.

Independent risk factors for previous HEV infection were being born in a nonwestern country and previous travel to subtropical and tropical regions, which can be explained by higher endemicity in nonwestern countries. Also, older age was a major risk factor for previous HEV infection, as observed by Sadik et al. (*17*).

A total of 9 of the 19 persons who showed seroconversion reported nonspecific symptoms possibly related to HEV infection. Only 1 of 31 hospitalized travelers showed seroconversion for antibodies against HEV, but hospitalization was probably not related to HEV infection (self-reported diagnosis was dehydration caused by diarrhea). None of the persons who showed seroconversion were given a diagnosis of HEV infection during the study. Because many cases of hepatitis E are subclinical in otherwise healthy persons and only immunocompetent travelers were included in our study, it is not surprising that so many persons who showed seroconversion did not report specific symptoms.

The strength of our study is that it is a prospective study in which blood samples before and after travel and diaries kept during travel were available for all 604 long-term travelers. However, our study also had limitations. Because this study was part of a larger study, the travel diary contained general clinical symptoms instead of hepatitis E–specific symptoms. Thus, we could have missed signs of a mild clinical HEV infection. Also, the median interval between obtaining a posttravel blood sample and return from travel was only 25 days. Because the incubation period for hepatitis E is 15–60 days, this period could have led to an underestimation of cases. However, because this study involved long-term travelers who traveled for 12–52 weeks, it is unlikely that many infections were contracted in the last weeks of travel. Therefore, we assume the short interval between return from travel and obtaining a blood sample had limited consequences for the final results. In addition, the median interval between obtaining the first blood sample and travel departure was 38 days. Therefore, persons who showed seroconversion might have contracted the virus before travel, which could have led to overestimation of travel-related attack rates and incidences, but we assume this had limited effect on the final results.

We assumed that persons with HEV IgG were protected against reinfection and did not include them in additional analyses. However, reinfection is possible, even in immunocompetent persons (*1*,*4*). We compared pretravel and posttravel sample titers and found only 1 person with a high posttravel titer and a much lower but still above the positive threshold pretravel titer. This result could have been a reinfection, but a low false-positive value for the pretravel sample is also possible. We considered this traveler immune in our additional analysis; this person also did not report any symptoms of HEV infection. A 4-fold increase in titer between pretravel and posttravel samples was not found for other persons.

Our study could have had a selection bias because all participants sought pretravel health advice in which advice on personal hygiene was stressed. Because genotypes 1 and 2 of HEV are contracted through the fecal–oral route, this finding could have led to an underestimation of HEV incidence. However, most (82%) travelers in our cohort experienced travelers’ diarrhea, which could also be contracted through the same route. Therefore, we believe that this selection bias had limited consequences on the outcome.

Finally, seroprevalence research most often lacks a diagnostic standard because it resembles a postinfection status in which conformatory tests using PCR are not feasible. In previous studies, the HEV IgG ELISA appeared to be one of the most sensitive tests available (*18**–**21*). However, in the absence of World Health Organization HEV-negative reference material, studies investigating the specificity of the test are scarce. Although results from a study in France were promising (specificity 97.8%) (*21*), possible false-positive test results cannot be excluded.

Using the HEV IgG ELISA, we found an attack rate for HEV infection of 3.7% and an incidence of 1.8 cases/1,000 person-weeks, which are higher than values from previous prospective studies. This finding could be a reflection of an increasing risk for travelers, but it could also (partially) reflect improved sensitivity of the available test. Almost half of persons who showed seroconversion had mild, nonspecific clinical symptoms possibly associated with HEV infection. Therefore, HEV infection does not seem to be a major problem in healthy immunocompetent travelers. However, rare fulminant liver failure in immunocompetent travelers has been reported (*2*), and in pregnant women and immunocompromised travelers, the risk for severe or fatal disease is much higher. Because travel has increased during the past few decades, at-risk groups also travel more (*22*). Good sanitation and clean drinking water should be discussed in all travel health advice. If an HEV vaccine were approved and found to be safe and effective for pregnant women and immunocompromised travelers, these vulnerable travelers could especially benefit from its protection.
